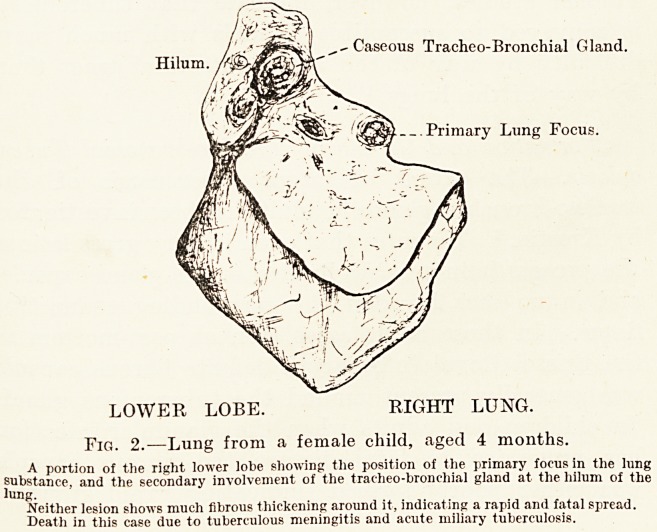# The Origin and Significance of Tuberculosis of the Tracheo-Bronchial Lymph Nodes of the Lung
*Awarded a Martyn Memorial Pathological Prize.


**Published:** 1930

**Authors:** L. R. Jordan


### THE ORIGIN AND SIGNIFICANCE OF TUBERCULOSIS OF THE TRACHEO-BRONCHIAL LYMPH NODES OF THE LUNG.*

BY

L. R. JORDAN.

---

### INTRODUCTION.

THE frequency of latent tuberculosis has been recognized since the latter part of the seventeenth century, but the true significance of these latent lesions in relation to other tuberculous conditions has not received the attention they merit. Recently, however, Continental and American workers have thrown some light on this important subject. In particular, E. L. Opie,[1] of Pennsylvania, points out the following two conflicting factors which must be considered when dealing with "latent tuberculosis."

1. The increased resistance to the disease from a somewhat ineffective immunization.

2. The danger that latent infection may cause manifest and perhaps fatal disease.

The significance of these factors with regard to latent disease will be discussed in the following remarks. The notes from 33 cases of tuberculosis examined post-mortem at the Bristol Royal Infirmary during the last two years, with either tracheo-bronchial or

* Awarded a Martyn Memorial Pathological Prize.

mesenteric infection associated with acute miliary tuberculosis, were used in preparation of this paper.

*Latent Tuberculosis.*—This may be defined as a tuberculous infection unaccompanied by significant symptoms evident to the patient or by physical signs discoverable by the physician.

The important question involved is, at what period of life does latent tuberculosis exist ? In the early years of childhood tuberculous infection nearly always proceeds to tuberculous disease, which is usually fatal, ending as acute miliary or acute pulmonary tuberculosis. Where the primary lesion is in the abdomen and thorax, for a short time, at least, there exists a "latent" period, during which no physical signs are discoverable at the routine clinical examination. Of the 26 cases of tracheo-bronchial and mesenteric tuberculosis at the Bristol Royal Infirmary only 3 cases at post-mortem revealed death due to causes other than tuberculosis, and the youngest was 7 years of age. MacNeil[2] reports a larger number of cases of latent tuberculosis, 18 in all: 1 case occurred under 2 years, 6 between 2 to 5 years, the remainder over 5 years, and he concludes that latent tuberculosis can scarcely be said to occur in infancy. Unfortunately MacNeil does not define his use of the word "latent," but he obviously means dormant or inactive tuberculosis. The definition given above may include active as well as dormant disease without physical signs, and the former, as will be seen, does exist during childhood, but is rarely recognized during life.

*First Infection with Tuberculosis.*—Tuberculosis in infancy and early childhood, which resembles that of an animal inoculated with tubercle bacilli, forms a lesion at the site of infection and quickly affects the nearest lymph nodes.

How far is this true in the human being? The commonest sites for lymph-node infection are, firstly, the tracheo-bronchial glands in the hilum of the lung, and, secondly, the mesenteric glands. In the latter case the lesion at the site of the infection occurs in the intestine or, as shown recently, may not form a visible intestinal lesion.

In the former case much controversy has taken place as to the site of primary infection. Some maintain that the lesion in the tracheo-bronchial nodes is primary. The majority of the evidence now seems to point to the lesion being secondary to a primary lesion in the periphery of the lung drained by the lymph nodes. When diligently looked for, a primary peripheral focus in the lung tissue has been shown to be present in the majority of cases.

Evidence in favour of this also supports the air-borne infection theory of tuberculosis, since the primary focus at the periphery of the lung occurs usually in a small bronchiole.

*Modes of Infection in the case of Tracheo-bronchial Tuberculosis.* — For the present purpose only two paths of infection need be considered, respiratory and alimentary. Both are equally probable in the child, the first from association with adult phthisical cases, the second by taking infected milk in its diet.

The belief that *infection through the respiratory tract* may occur is supported by the following evidence :—

1. In anthracosis and allied diseases coal and other dusts can be inhaled deeply into the lungs.

There is no reason why bacilli should not be inhaled in droplets or dust particles and give rise to lesions in the lung.

2. Guinea-pigs exposed to dust or spray laden with tubercle bacilli were found on necropsy, some time later, to show lesions in the most distant bronchioles and alveoli, and tubercle bacilli were recovered from these parts.

3. Ghon,[3] and more recently MacCallum[3] demonstrated in every case of tuberculosis of the lungs in children the presence of an old primary lesion in the extreme periphery of the lung followed by lymphatic transportation to the bronchial nodes. Kuss[4] who also found the nodule in almost every case, and Opie who confirmed the results, regarded "the consistence and nature of the nodules as evidence of considerable age and typical of lesions sustained during childhood." Canti[5] in his own cases states that the changes in the glands never appeared older than those in the lung focus. He also regards it as proved "that tuberculous tracheo-bronchial glands are not secondary to a focus anywhere else in the body."

Recent work by Miller on the pulmonary lymph flow makes it difficult to accept the view that a retrograde infection from the hilum to lung tissue can occur.[6] Krause, however, has shown experimentally that organisms may pass through the lung tissue, leaving the latter unaffected, and produce the primary lesion in the lymph nodes.[6] This is probably very rare in the human being, but it may account for rare cases of lymph-node infection which do not reveal a primary lesion on careful examination.

Of the 20 cases of tracheo-bronchial tuberculosis seen at routine post-mortem examinations 5 showed definite primary foci in the periphery of the lung. In the remaining 15 cases the lesions were most probably present, but overlooked for the following reasons :—

1. No special search was being made.

2. The nodules can be very minute, and without careful dissection not easily found. Krestin[6] remarks that X-ray films of the excised inflated lungs are a great aid to their detection.

3. In adults showing hilum infection the primary lesions were most probably healed and represented by a small scar.

4. The primary lesion may be masked by co-existent miliary tuberculosis, or by tuberculous broncho-pneumonia and secondary septic conditions which are present in a majority of cases.

The 5 cases in which the primary foci in the lung tissue were well marked and easily discovered were all in children under 7 years, and the lungs in these cases did not show any other gross obscuring lesions. Two specimens were kept showing tracheo-bronchial tuberculosis of the lymph nodes and primary foci in the lung tissue.

**Case 1.**—Male, aged 7, whose left lung showed the following lesions. (Fig. 1.)

At the apex of the upper lobe of the left lung there was a patch of quiescent tuberculosis ; this was the primary focus. It consisted of two caseous nodules surrounded by a definite fibrous thickening, measuring about 2 cm. by 1 cm. A second smaller caseous nodule was visible under the pleural surface some distance away. At the hilum is one enlarged caseous " sentinel " gland at the upper part, which corresponds to the gland draining the infected area. There are some other enlarged hyperæmic glands in the hilum with no sign of caseation, they are probably early tuberculous. The primary lesion appears to be much older than the glandular infection, as judged by the degree of fibrosis and caseation.

This case did not show any military tuberculosis. Death was due to another cause, acute myocarditis and subacute endocarditis.

**Case 2.**—Female, aged 4 months. (Fig. 2.) The lesion progressed rapidly and ended in tuberculous meningitis with acute miliary tuberculosis of the spleen and kidney.

The right lung, lower lobe, showed a small caseous area with small cavities. A caseous gland at the hilum, corresponding to the area drained by lymphatics, was present, and showed an area of recent spread into the lung. There was practically no fibrosis, showing that the lesion had progressed rapidly, entered the blood-stream and scattered as miliary tuberculosis throughout the body.

FIG. 1.—Lung from a male child, aged 7 years.

The cut surface shows a large primary focus in the upper lobe; it consists of two caseous nodules surrounded by a definite fibrous thickening. Measures 2 cm. by 1 cm.

The hilum shows an encapsulated caseous tracheo-bronchial gland and several enlarged and hyperæmic glands cut through. These show no caseation, but one probably tuberculous, being recently infected by lymphatic or direct spread from the other gland.

A small caseous nodule is also seen on the pleural surface of the upper lobe.

Both the primary focus and the tracheo-bronchial gland show a degree of fibrosis not seen in those rapidly growing lesions ending at a much earlier age in acute miliary tuberculosis.

Death in this case due to acute myocarditis and subacute endocarditis.

*The alimentary route* in infection of tracheo-bronchial glands has not so much evidence in favour of it as the respiratory. Nevertheless, lungs which do not show a primary lung tissue lesion in tracheo-bronchial tuberculosis may possibly be infected by means of the lymphatics or blood-stream from the alimentary canal. This would seem to account for the bilateral infection of tracheo-bronchial lymph nodes, which does occur without any easily-found primary focus.

Usually the majority of lesions in tracheo-bronchial tuberculosis occur on the right side. In this series 8 cases out of 14 cases of tuberculosis were right-sided, 4 being left-sided and 2 occurring on *both* sides. (Six of the cases had to be excluded, as the notes did not state definitely on which side the lesions occurred.) These figures agree with Wollestein's, who found 74 per cent. lesions on the right side. [8]

FIG. 2.—Lung from a female child, aged 4 months.

A portion of the right lower lobe showing the position of the primary focus in the lung substance, and the secondary involvement of the tracheo-bronchial gland at the hilum of the lung.

Neither lesion shows much fibrous thickening around it, indicating a rapid and fatal spread. Death in this case due to tuberculous meningitis and acute miliary tuberculosis.

Even in the case of those lungs which show a primary focus in the lung tissue, the possibility of the original bacilli reaching the lungs by the circulation, and there forming a tubercle, with subsequent lymph-borne infection of tracheo-bronchial glands, cannot be excluded in every case.

Von Behring[3] points out that in very young infants the ferment-secreting glands of the digestive tract are little developed and the epithelial lining not yet a serious obstacle to the passage unchanged of any foreign protein. A rapid general spread of such bacteria throughout the body, lodging in the lymphoid tissue everywhere, is usual without any lesion in the intestinal mucosa.

Experimentally, pigment given to animals by mouth finds its way from the alimentary canal and collects in the lungs and bronchial glands, showing that the latter have some peculiar filtering effect. Further studies, however, indicate that tuberculosis is conveyed to susceptible animals with much more difficulty by way of the gastro-intestinal tract than by way of the lungs.[1]

*Formation and Growth of Tracheo-bronchial Tuberculosis.*—The extent of latent tuberculosis of the tracheo-bronchial glands in adults has been investigated by Krestin.[6] He notes that even in very gross lesions the process is invariably limited to the gland capsule, and in no case invaded the surrounding connective tissue. In those adult cases seen at post-mortem in the Bristol Royal Infirmary a definite fibrous capsule was generally present around the tuberculous gland. In children and adults when the glandular infection has proceeded to fatal disease the fibrous capsule was either poorly formed (indicating rapid growth), or recent spread through the neighbouring tissue was observed.

The growth of tracheo - bronchial tuberculous lymph nodes is rapid and destructive, especially in the young child. Except when latent tracheo-bronchial tuberculosis is present the lymph nodes at the hilum are not conspicuously affected in the adult. Apparently there has been time for resistance to develop to such a degree that the destruction of tissue is held in check. The infant does not develop this resistance until it has reached several years of age, and it increases with the age of the child.

Tracheo-bronchial lesions after the early years tend more and more to heal and become latent. The majority of cases before 2 years of age, however, end rapidly in acute miliary or acute broncho-pneumonic tuberculosis.

Those cases which in later years of life become the "latent" lesions may or may not be the cause of more deadly disease in the adult. In the cases studied (9 cases of acute miliary tuberculosis between the ages 12–25 years) 6 cases showed tracheo-bronchial and 1 case mesenteric tuberculosis, and the remaining 2 cases were secondary to chronic phthisis. The remaining cases of tracheo-bronchial tuberculosis either end in tuberculous broncho-pneumonia (3 cases) or remain latent.

The earliest case in the series of tracheo-bronchial tuberculosis was a child of four months, probably the earliest recorded. The only other case under one year was a child aged 10 months, who showed tracheo-bronchial tuberculosis with miliary tubercles in the lung, pleura, liver and spleen. There was no meningitis in this case. Krause,[7] in experiments on animals, has recently shown that tracheo-bronchial gland

R

infection can take place in non-immune animals in from seven to forty days. In this case the presence of tubercle bacilli in the glands was demonstrated by injecting them into other guinea-pigs. In the child of 4 months infection has obviously occurred soon after birth, but in the majority of children in this series death occurred during the second year of life. Opie,[1] working on much larger figures, says that the first year of life shows the greatest mortality, followed by a gradual decline to the fifth year and over. He comes to the conclusion that tuberculous infection in childhood increases resistance to the disease, but is at the same time a source of danger.

*Acute Miliary Tuberculosis.*—As we have seen, the most likely result of tracheo-bronchial tuberculosis is acute miliary tuberculosis. It occurs as a fatal termination to those cases of rapid infection during childhood, or to previously latent disease at almost any age. In this series 75 per cent. of cases of acute miliary tuberculosis between birth and 5 years and 59 per cent. of these between 12–40 years of age showed tracheo-bronchial infection. According to the figures it is nearly six times as common during childhood (1–5 years) as in the adult (12–40 years), and a considerable number of cases are associated with tuberculous meningitis.

Of 29 cases at all ages in the series, 60 per cent. showed tuberculous meningitis, 45–57 per cent. miliary tubercles in the pleura or lung, 63 per cent. in the spleen, 57 per cent. in the liver and kidneys, and 25 per cent. in the peritoneum.

Tuberculous meningitis is thus the commonest form of death in the acute tuberculous diseases. Miliary tubercles of pleura and lung were present in a large number of cases, four of which showed involvement of the lung only. In one case the pleura alone was involved. Of the abdominal organs the spleen was the most and the liver and kidneys less frequently involved. The lesions in the liver were chiefly subcapsular, miliary tubercles rarely being found in the liver substance. The peritoneum was only involved in a comparatively few cases. Miliary tuberculosis of the lung alone was not seen ; the cases, 16 in number, were all associated with miliary tubercles of the other organs, and 10 of these cases showed meningitis.

Of the 17 cases showing tuberculous meningitis all were associated with miliary tuberculosis of some other organ or organs.

In 3 cases of tuberculous meningitis no primary lesion was discovered at the routine post-mortem examination. These, the only cases of acute miliary tuberculosis in which this occurred, were, curiously enough, all females.

On the other hand, it may lend support to the theory, held by some, that this disease is sometimes primary in a small number of cases which fail to reveal primary lesions even on the most careful investigation.

The following table gives some idea of the distribution of such primary lesions as was found in the series :—

| Age. | Bristol Figures. | | | MacNeil's. | | |
|---|---|---|---|---|---|---|
| | Pulmonary. | Abdominal. | Cervical. | Pulmonary. | Abdominal. | Cervical. |
| Birth—2 years.. | 1 | 1 | 0 | 13 | 10 | 1 |
| 2—5 years .. | 4 | 1 | 0 | 9 | 4 | 0 |
| 5—12 years .. | 1 | 0 | 0 | 11 | 2 | 1 |
| Total .. .. | 6 | 2 | 0 | 33 | 16 | 2 |

Over 12 years of age the commonest period in the series of meningitis with tracheo-bronchial tuberculosis was from 15 to 25 years. Although from a much smaller number of cases, it is obvious from the above table that the majority of "primary" lesions occur in the chest (*i.e.*, tracheo-bronchial) from 2–5 years, showing a rise after the first two years, instead of a fall as MacNeil's figures show.

Comparing the figures of the abdominal cases, it will be seen that, apart from the difference in numbers, they correspond as far as age incidence is concerned.

Though these small figures count for little, in this case they are probably borne out by larger ones, and the results may be put down to a difference in locality from which the cases were drawn.

Apart from the above-mentioned differences, there is ample evidence to support the general statement "that nearly all cases of tuberculous meningitis are derived from a glandular or visceral focus in the thorax or abdomen, a clear majority being thoracic."

It is uncommon for tuberculous meningitis to develop from glandular disease of neck and throat. No case was found in the series. The fact that all the cases of tuberculous meningitis were associated with the miliary tubercles in other organs indicates a blood-borne infection in every case. The possibility of a lymphatic or direct spread causing the meningitis seems very remote, and does not explain the cases in which no primary focus was discovered.

*Recognition of Tracheo-bronchial Tuberculosis during Life.*—This at present is a difficult matter. In none of the cases in the series was the presence of tracheo-bronchial tuberculosis recognized clinically, although the majority ended fatally as acute generalized tuberculosis when it was too late for any interference. Opie[1] and others, by the use of the von Pirquet reaction and X-ray films of the chest, claim to have discovered a large number of cases of tracheo-bronchial tuberculosis during life. It has been found by this means that this disease is seven times more common in children exposed to massive infection from active phthisical cases in the family. Conversely, the discovery at post-mortem of tracheo-bronchial tuberculosis in children ending fatally may be a clue to the presence of active phthisis in one of the adults of the family, hitherto undiscovered. MacNeil[2] points out that a positive tuberculin test with or without positive skiagrams in children in early years is of grave significance, as the disease is rarely "latent" at this age, and active tuberculosis, as we have seen, is generally fatal.

### Conclusions.

1. The majority of evidence at present points to tuberculous infection of the tracheo-bronchial glands being secondary to a primary air-borne infection in the lung tissue.

2. Tuberculosis, especially of the tracheo-bronchial glands in children under 5 years, is not dormant, but in the majority of cases ends in fatal disease.

3. A considerable proportion of acute miliary tuberculosis, especially with tuberculous meningitis, in adults has its origin in tracheo-bronchial tuberculosis.

4. All the cases of tuberculous meningitis were blood-borne, and no explanation is offered for the absence of visible primary foci in these cases.

5. Tuberculous infection in childhood increases resistance to the disease, but at the same time is a source of danger.

I should like to thank Dr. Fraser for permission to use the post-mortem notes and pathological material, and for the encouragement he has given me in the preparation of this paper.

### REFERENCES.

[1] Opie, E. L., "The Pathology of Tuberculosis in Childhood and its bearing on Clinical Work," *Brit. Med. Jour.*, 1927, ii. 1,130.

[2] McNeil, C., "Tuberculosis in Early Childhood," *Brit. Med. Jour.*, 12th October, 1929, p. 655.

[3] MacCallum, W. G., *Textbook of Pathology* (fourth edition), p. 599.

[4] Kuss, *De l'heredite parasitaire de la tuberculose humane*, Paris, 1898.

[5] Canti. (In discussion following Opie's "Pathology of Tuberculosis in Childhood," *Brit. Med. Jour.*, 1927, ii. 1,130.)

[6] Krestin, D., "Latent Tuberculosis Lesions in the Lungs as determined by Roentgenological and Pathological Investigation," *Quart. J. Med.*, April, 1929, No. 87, p. 541.

[7] Burrell, L. S., *Recent Advances in Pulmonary Tuberculosis*, p. 5.

[8] MacCrae, T., Osler's *Principles and Practice of Medicine*, 1927, p. 178.

## Figures and Tables

**Fig. 1. f1:**
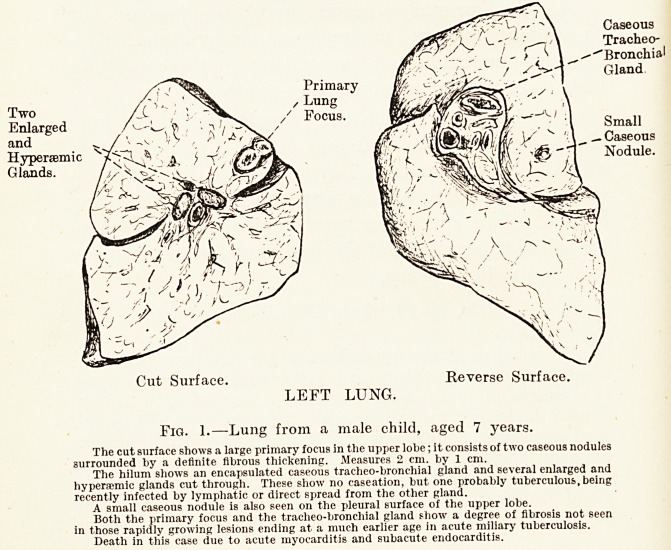


**Fig. 2. f2:**